# Epigenetic silencing of the non-coding RNA nc886 provokes oncogenes during human esophageal tumorigenesis

**DOI:** 10.18632/oncotarget.1927

**Published:** 2014-04-27

**Authors:** Hyun-Sung Lee, Kwanbok Lee, Hee-Jin Jang, Geon Kook Lee, Jong-Lyul Park, Seon-Young Kim, Sang-Bae Kim, Betty H. Johnson, Jae Ill Zo, Ju-Seog Lee, Yong Sun Lee

**Affiliations:** ^1^ Department of Systems Biology, The University of Texas MD Anderson Cancer Center, Houston, TX 77030, USA; ^2^ Center for Lung Cancer, Research Institute and Hospital, National Cancer Center, Goyang, 410-769, Korea; ^3^ Department of Biochemistry and Molecular Biology, The University of Texas Medical Branch, Galveston, TX77555-1072, USA; ^4^ Medical Genomics Research Center, KRIBB, Daejeon, 305-806, Korea; ^5^ Department of Functional Genomics, University of Science and Technology, Daejeon, 305-806, Korea; ^6^ Department of Thoracic and Cardiovascular Surgery, Samsung Medical Center, Sungkyunkwan University School of Medicine, Seoul, Korea; ^7^ Department of Biochemistry and Molecular Biology, Medical Research Center and Biomedical Science Institute, School of Medicine, Kyung Hee University, Seoul 130-701, Korea.

**Keywords:** nc886, ESCC, CpG DNA methylation, tumor suppressor

## Abstract

nc886 (= vtRNA2-1 or pre-miR-886) is a recently discovered noncoding RNA that is a cellular PKR (*P*rotein *K*inase *R*NA-activated) ligand and repressor. nc886 has been suggested to be a tumor suppressor, solely based on its expression pattern and genomic locus. In this report, we have provided sufficient evidence that nc886 is a putative tumor suppressor in esophageal squamous cell carcinoma (ESCC). In 84 paired specimens from ESCC patients, nc886 expression is significantly lower in tumors than their normal adjacent tissues. More importantly, decreased expression of nc886 is significantly associated with shorter recurrence-free survival of the patients. Suppression of nc886 is mediated by CpG hypermethylation of its promoter, as evidenced by its significant negative correlation to nc886 expression in ESCC tumors and by induced expression of nc886 upon demethylation of its promoter. Knockdown of nc886 and consequent PKR activation induce FOS and MYC oncogenes as well as some inflammatory genes including oncogenic NF-κB. When ectopically expressed, nc886 inhibits proliferation of ESCC cells, further demonstrating that nc886 could be a tumor suppressor. All these findings implicate nc886 as a novel, putative tumor suppressor that is epigenetically silenced and regulates the expression of oncogenes in ESCC.

## INTRODUCTION

We have recently identified a 101 nucleotide (nt) long non-coding RNA (ncRNA), nc886 (also prematurely named as vtRNA2-1 or pre-miR-886), that is ubiquitously expressed in normal tissues. nc886 could be a tumor suppressor as suggested by several lines of observations. First, its expression is decreased in cancer cell lines from several tissue origins [1, 2]. Second, a CpG island at its promoter region is hypermethylated in lung cancer and acute myeloid leukemia [3, 4]. Third, its genomic locus at human chromosome 5q31 is frequently deleted in leukemia [5, 6].

Thus far, nc886's known function is a cellular RNA ligand and inhibitor of PKR (*P*rotein *K*inase *R*NA-activated), a pleiotropic protein implicated in cellular defense against virus, stress responses, inflammation, and tumorigenesis (reviewed in [7]). Knockdown of nc886 activates PKR and ectopic expression of nc886 represses the interferon response triggered by double-stranded RNA (dsRNA), a canonical PKR activating ligand [1, 2, 8]. Presumably, nc886's function in normal cells is to adjust a cellular level of tolerance to diminutive triggers which should be normally insignificant so that signaling pathways and metabolism are not disturbed. However, its molecular roles in tumor development are currently unknown.

Tumorigenesis is a multi-step process driven by genetic/epigenetic alterations causing activation of oncogenes as well as inactivation of tumor suppressor genes. Activation of oncogenes can be driven also by extracellular signals or environmental cues. For instance, the expression of FOS and MYC oncogenes are induced by growth factors [9], oxidative stress [10], dsRNA, and viral infection [11]. Another example is oncogenic NF-κB, whose activation in cancer is attributed mostly to pro-inflammatory stimuli, but rarely to genetic/epigenetic alterations (reviewed in [12]).

Esophageal cancer (EC) is one of the most malignant and dismal prognostic tumors, ranked eighth in incidence rate and sixth in cancer-related death worldwide [13]. EC is classified into two major histopathological types: esophageal squamous cell carcinoma (ESCC) and esophageal adenocarcinoma (EAC). These two subtypes differ in carcinogenesis, cancer genetics, prognosis and pattern of recurrence [14]. ESCC is dominant over EAC worldwide. In case of EAC, its pre-malignant stage is metaplasia such as Barrett's esophagus which is most likely caused by chronic exposure to acid and bile reflux. However, the molecular mechanism of ESCC carcinogenesis is still elusive, except for some information that its etiology is correlated with smoking and consumption of hot tea. Furthermore, lack of good diagnostic markers and treatment strategies has rendered ESCC a major challenge in clinic. As an endeavor to understand ESCC, we investigated nc886 in this study.

## RESULTS

### Suppression of nc886 expression in ESCC is caused by CpG hypermethylation

**Figure 1:**
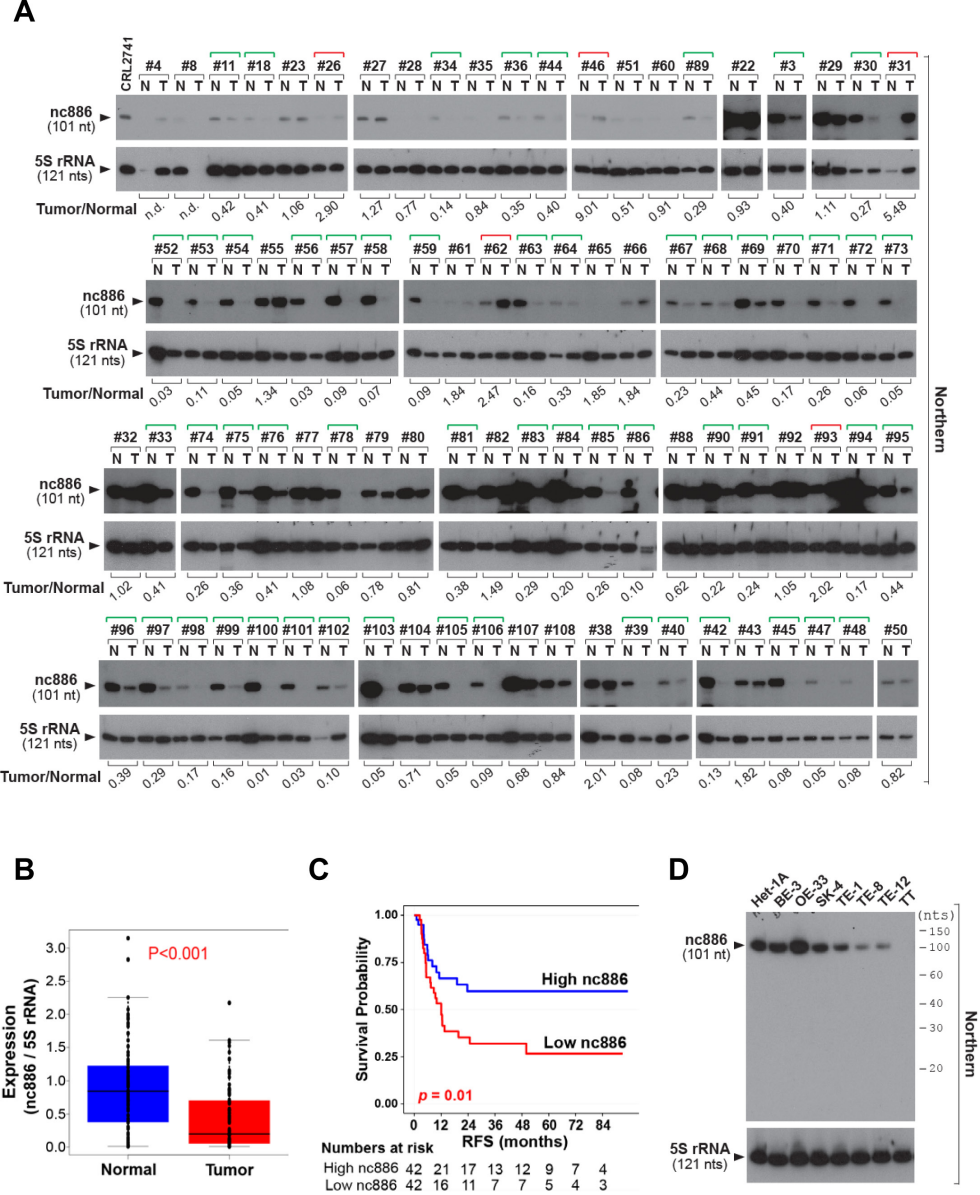
nc886 expression suppression in clinical specimens from ESCC patients and esophageal cell lines A. Northern hybridization of nc886 and 5S rRNA (for equal loading) in 84 pairs of an ESCC tumor and its adjacent normal tissue (designated T and N respectively). Sample identity is anonymously designated in #-number on the top of gels. RNA from a nc886 expressing cell line CRL2741 was included as a quality control [2]. Each band was quantified with AlphaView software 2.0.1.1 (Alpha Innotech, Santa Clara, CA). nc886 values were normalized to 5S rRNA values, and nc886/5S rRNA values of each tumor relative to its normal tissue is shown at the bottom (“Tumor/Normal”). “n.d.” indicates “not determined” because 5S rRNA values could not be obtained due to RNA degradation. Green and red brackets on the top designate “Tumor/Normal” value less than 0.5 and more than 2, respectively.**B**. Comparison of nc886/5S rRNA values (from panel A) between tumors and adjacent normal tissues.**C**. Recurrence-free survival (RFS) curve. The 84 patients were classified into two sub-groups according to nc886 RNA expression levels (the “Tumor/Normal” value in panel A). High- and low- nc886 group was discriminated by the median value. Patients at risk were added below the survival curve.**D**. Northern hybridization of nc886 and 5S rRNA as a loading control in esophageal cell lines. Molecular sizes from Decade markers (= 10-nt ladder) are indicated on the right.

As the first step to investigate nc886 in EC, we measured the expression of nc886 in 84 pairs of a tumor tissue and its adjacent normal tissue from ESCC patients. nc886 expression was significantly suppressed in tumors (Fig 1A-B). It is worth pointing out that our measurement was done by Northern hybridization to ensure detection of nc886 as a 101 nt long ncRNA. Mature microRNA (miRNA) was detected in none of the samples (Fig S1). When the ESCC patients were stratified according to nc886 expression, its lower expression was significantly (P = 0.01) correlated with shorter recurrence-free or overall survival of the patients (Fig 1C and S2). Clinico-pathological characteristics of the nc886 high- and low- expression groups were summarized in Table S1. nc886 expression was also decreased in ESCC cell lines (TE-1, TE-8, TE-12, and TT) relative to a nonmalignant esophageal cell line Het-1A (Fig 1D). Of note, nc886 expression in Barrett's esophagus, metaplasia and EAC cell lines (BE-3, OE-33 and SK-4 respectively) remained as high as in Het-1A (Fig 1D).

**Figure 2:**
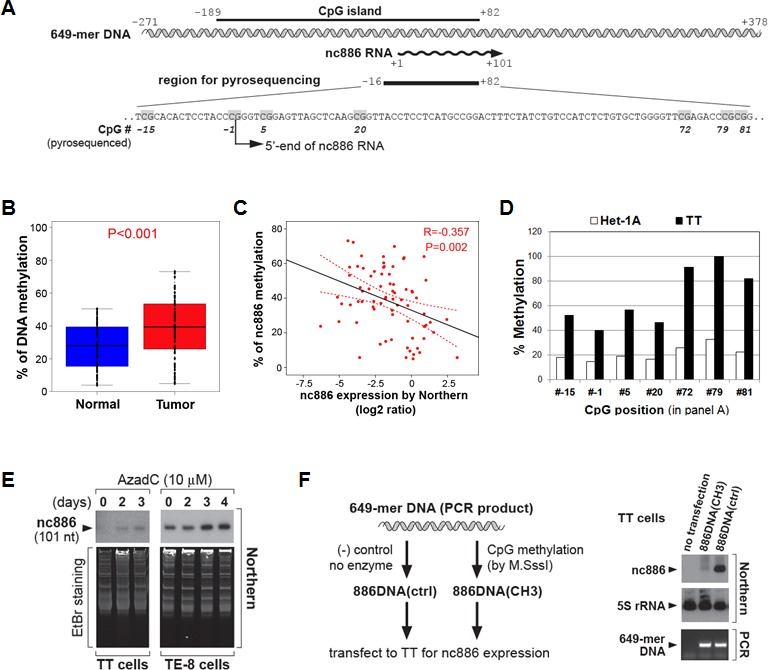
CpG DNA methylation of the nc886 promoter region as a cause for suppression of nc886 RNA expression A. Diagram depicting a nc886 genomic region encompassing 649 nts (illustrated in double helix). All nt coordinates are numbered referring to nc886 transcript's 5'-end nt as +1. Pyrosequenced CpG dinucleotides are indicated in grey.**B**. Comparison of nc886 CpG methylation levels between tumors and adjacent normal tissues. Among the 84 patients in Fig 1, 73 patients yielded genomic DNA with adequate quality and quantity, and thus could be examined by pyrosequencing. The seven CpG dinucleotides (grey highlighted in panel A) were assayed for percent methylation and their average values plotted (y-axis).C. Spearman correlation analysis between nc886 RNA expression (from Fig 1A-B) and CpG methylation (from panel B). **D**. Percent methylation of individual CpG dinucleotides (in panel A) in Het-1A and TT cell lines. E. Northern hybridization of nc886 with ethidium bromide (EtBr) staining shown for equal loading, after treating cells with 10 μM of AzadC for indicated days.F. Transfection of an *in vitro* methylated nc886 DNA fragment. Left panel: the experimental scheme. Right panel: Northern hybridization of nc886 and 5S rRNA, together with PCR measurement of transfected DNA to confirm equal transfection efficiency between “886DNActrl” and “886DNA(CH3)”. For more details, see Supplemental Information.

Our inspection of the nc886 genomic region (by using http://cpgislands.usc.edu/, [15]) detected a strong CpG island (Fig 2A). Our pyrosequencing data in the ESCC patient samples indicated that the nc886 promoter region tended to be hypermethylated in tumors compared to adjacent normal tissues (Fig 2B). This CpG hypermethylation was a cause of nc886's suppressed expression in ESCC, as evidenced by the following data. First, negative correlation was seen between CpG DNA methylation and RNA expression in the ESCC tumors and cell lines (Fig 2C-D). Second, treatment with 5-Aza-2'deoxycytidine (AzadC), a DNA methyltransferase inhibitor, led to de-repression of nc886 expression in TT and TE-8 cells (Fig 2E). Third, nc886 transcription was active from a transfected DNA fragment (649-mer DNA shown in Fig 2A) but was inactivated when the DNA fragment was in vitro methylated (Fig 2F).

### Induction of oncogenes upon nc886 knockdown

**Figure 3:**
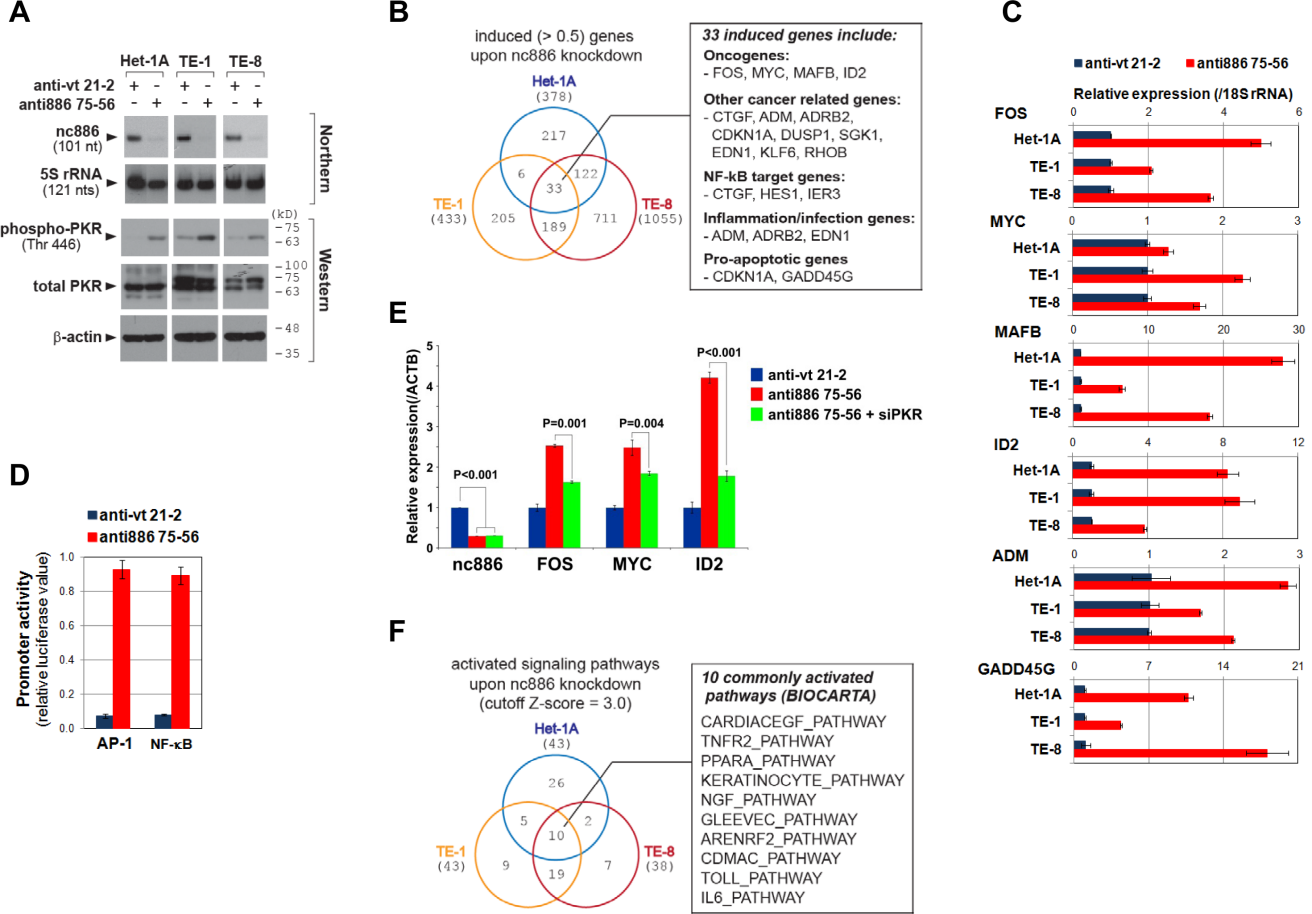
Gene expression profile and pathway analysis after nc886 knockdown A. Northern hybridization and Western blot of indicated RNA and proteins after nc886 knockdown. Molecular sizes in kilodalton (kD) from the size marker are indicated on the right for Western blot. **B**. Venn diagram of induced genes upon nc886 knockdown in the three cell lines. 33 commonly induced genes were further scrutinized and summarized in a box on the right.**C**. qRT-PCR measurement of selected genes from the 33 genes. Ct values of each gene were converted to relative quantity (2^−Ct^) and normalized to 2^−Ct^ values from 18S rRNA. Values of “anti-vt 21-2” were set as 1 (blue bars). An average and the standard deviation from triplicate experiments are indicated.**D**. Luciferase assay to determine the activity of AP-1 and NF-κB target promoters. These promoters are activated by AP-1 and NF-κB and drive expression of firefly luciferase gene (“*Pp*”). Each *Pp* reporter plasmid was transfected into TE-8 cells together with pRL-SV40, a plasmid expressing renilla luciferase (“*Rr*”). *Pp* values were first divided by *Rr* values to normalize transfection efficiency, and then *Pp*/*Rr* values for AP-1 or NF-κB were again normalized to *Pp*/*Rr* values from pcDNA3-1 Zeo(+)-*Pp*, an irrelevant *Pp* plasmid. Values from “anti-vt 21-2” were set as 1. An average and the standard deviation were calculated from triplicate experiments.**E**. qRT-PCR measurement of FOS, MYC and ID2 after nc886 and PKR knockdown. Anti-oligos and siRNA (against PKR) were co-transfected into TE-8 cells as previously described [1]. Except that ACTB was used for normalization, all other descriptions are the same as panel C.**F**. Venn diagram of activated signaling pathways (BIOCARTA pathways) analyzed from the mRNA array data upon nc886 knockdown in the three cell lines.

To explore cellular events triggered by nc886 suppression, we examined global gene expression profiles in Het-1A and two ESCC cell lines (TE-1 and TE-8) by mRNA array after nc886 knockdown. Efficient knockdown was confirmed by Northern hybridization (representative Northern blots shown in Fig 3A) and by our array data in which nc886 was the most decreased gene in the three cell lines (Fig S3).

From gene expression data, we sorted genes according to fold-change and extracted a set of the most increased (or decreased) genes. For example, 378, 433, and 1055 genes were selected as significantly induced (P < 0.01 and higher than 0.5-fold) genes respectively from Het-1A, TE-1, and TE-8 cells, when nc886 expression was silenced (Fig 3B). While 33 genes were induced in all three cell lines, only 6 genes were commonly repressed (Fig S4).

Therefore, we focused on induced genes, especially the 33 commonly induced genes (Fig S3 and Table S2). More than one third were cancer-related (Fig 3B) according to classified cancer genes in Cancer Portal (http://rgd.mcw.edu/wg/portals/). Notably, the 33 genes included well-known oncogenes FOS, MYC, MAFB, and ID2, all of which have been shown to have a transforming ability when aberrantly expressed [16-19]. Their induction in the array data was confirmed by qRT-PCR measurement (Fig 3C). FOS encodes a subunit of the activator protein-1 (AP-1) and MAFB is also a member of the AP-1 family. In accordance with the induction of FOS and MAFB expression, AP-1 activity was elevated as proven by our reporter assay in which luciferase expression was driven by a promoter containing AP-1 recognition elements (Fig 3D). Oncogenic NF-κB was also activated, as shown by its target genes in the 33 genes (Fig 3B) and by elevated luciferase expression whose promoter contained NF-κB target sites (Fig 3D).

FOS and MYC have been classically known as “immediate early genes” that surge quickly upon growth stimuli and then decline [9]. Interestingly, these genes are also induced by dsRNA [11], which is a viral replication intermediate and the best ligand for PKR activation. This induction is known to be abrogated by 2-aminopurine, an inhibitor for kinases including PKR [20]. It is also known that active PKR provokes the NF-κB pathway (reviewed in [7]). Our previous finding was that nc886 is a PKR inhibitor [1, 2, 8]. In our data here, nc886 knockdown activated PKR in the three cell lines, as shown by the appearance of phospho-PKR, an active form of PKR (Fig 3A). The induction of FOS, MYC and ID2 upon nc886 knockdown was significantly mitigated by siRNA-mediated knockdown of PKR (Fig 3E). All these data clearly indicated that these oncogenes were activated through the nc886-PKR pathway and suggested nc886 as a tumor suppressor in ESCC.

nc886 knockdown also induced inflammation/infection genes and pro-apoptotic genes (Fig 3B). This was not surprising, given that PKR was activated (Fig 3A). As PKR activation typically occurs during viral assault, cells would have responded to nc886 knockdown as if virus infected and were committed to death before exhibiting any malignant phenotype (data not shown). This was corroborated by activation of the Toll-like receptor pathway (“TOLL_PATHWAY” in Fig 3F) in our pathway analysis. As in a cellular response to pathogen or stress, nc886 knockdown provoked many signaling pathways and consequently induced transcription factors (Fig S5). Intersection of activated pathways in the three cell lines exhibited a significant overlap and yielded ten common pathways (Fig 3F). All the ten pathways involved AP-1, MYC, and NF-κB, in concordance with the increased expression of FOS, MAFB, and MYC.

**Figure 4:**
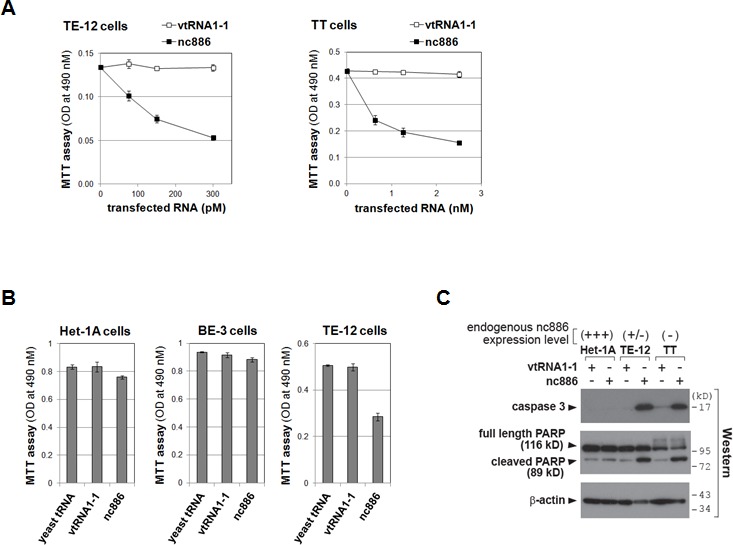
Impaired cell proliferation of ESCC cells upon ectopic expression of nc886 A-B. Cell proliferation (MTT) assays at 24 hrs after transfection of *in vitro* transcribed nc886 or vtRNA1-1 at indicated concentrations (panel A) or after transfection of indicated RNAs at 150 pM (panel B). Averages and standard deviations were calculated from triplicate samples.C. Western blot of indicated proteins at 24 hrs after transfecting 150 pM of nc886 or vtRNA1-1. β-actin is shown for equal loading.

Since our data so far indicated that nc886 is a putative tumor suppressor in ESCC, we questioned whether re-expression of nc886 in ESCC cells would render any anti-proliferative and/or pro-apoptotic phenotype. To test this, we sought to construct a transgenic TT cell line stably expressing nc886. Despite multiple attempts in TT and also another cell line TE-12, we failed to recover those cells, indicating that nc886 expression was deleterious in ESCC cells. So, we made nc886 RNA by *in vitro* transcription and transfected it as an alternative way to assess nc886's acute effect. When transfected at sub-nanomolar levels, nc886 RNA significantly inhibited proliferation of TE-12 and TT cells (Fig 4A), both of which are ESCC cells expressing very low levels of nc886 (see panel C and also Fig 1D for their nc886 expression levels). In contrast, the same treatment did not inhibit proliferation of non-ESCC cells where nc886 expression was not suppressed (Het-1A and BE-3 cells in Fig 4B). We further measured apoptotic markers (caspase-3 and PARP cleavage in Fig 4C) and found that nc886 induced apoptosis in the ESCC cells but not in Het-1A cells. Our data proves nc886's potent and selective pro-apoptotic activity, in agreement with its tumor suppressor role in ESCC.

## DISCUSSION

**Figure 5:**
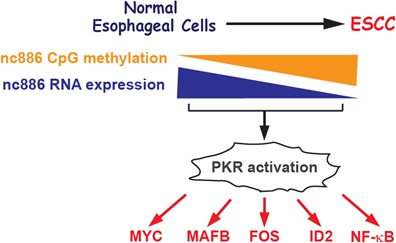
Cartoon summarizing results in this study

In this report, we obtained several lines of evidence supporting that nc886 is a putative tumor suppressor in ESCC. First, nc886 expression was significantly decreased in ESCC tumors by CpG hypermethylation at its promoter, a common mechanism to silence tumor suppressor genes. Second, the lower expression of nc886 was associated with poorer survival of ESCC patients. Third, nc886 knockdown activated oncogenes. Forth, re-introduction of nc886 inhibited the growth of ESCC cells. Our results are summarized in Fig 5.

It is very important to point out that nc886's activities and features in ESCC were represented by its full length in size of 101 nts, but not by its miRNA product (miR-886). We have not detected miR-886 in our Northern blots. Also, our anti-oligo for nc886 knockdown was off-target from miR-886. Furthermore, miR-886 has been removed from the miRNA database (miRbase: www.mirbase.org). We infer that nc886's central portion is more important than either ends harboring mature miR-886-5p or -3p, because the central portion is the binding domain for PKR [8] and PKR activation was a reason for the induction of several oncogenes upon nc886 knockdown (Fig 3).

A striking result in this study was that nc886 knockdown induced many genes including the renowned oncogenes MYC and FOS (Fig 3). These genes do not share any sequence homology to nc886, and so nc886's regulatory action on these genes could not be through a miRNA mechanism (reviewed in [21]). Recently, nuclear ncRNA's role in regulation of gene expression through chromatin remodeling has been intensively studied (reviewed in [22]); however, this mechanism was not likely either because nc886 is exclusively in the cytoplasm [2]. Based on our data regarding PKR (Fig 3A and E), it would be most reasonable to interpret that nc886 knockdown was a mimicry of viral infection and accordingly induced MYC and FOS as well as genes related to inflammation and infection such as oncogenic NF-κB. The causal relationship between inflammation and cancer is well documented from many studies and widely accepted (reviewed in [23]).

Our data also opens a possibility to use nc886 in clinical applications. nc886 RNA expression and/or its DNA methylation can be a prognostic marker for ESCC patients. nc886 RNA level is very abundant and easier to measure than miRNAs [2], because it is 101 nts long and so can be measured by regular qRT-PCR with two specific primers. Measurement of its CpG methylation can be a proxy marker, if RNA quality or tissue contamination is a concern. Detection of nc886 depletion by methylation in ESCC might classify the high risk patients with recurrence and identify candidates for therapy with peri-operative adjuvant treatment. Because patients with reduced nc886 expression would have earlier recurrence than others, it would be advisable to utilize more aggressive treatments such as chemotherapy or chemo-radiation after surgery. However, this approach needs to be tested in a prospective clinical study. Also, nc886's selective toxicity to ESCC cells (Fig 4) could be utilized in cancer treatment in the future.

Our work here is the first extensive study of nc886 in ESCC. Our expression and methylation data from copious patient samples indicate nc886's clinical significance. We speculate that epigenetic silencing of nc886 is a cell autonomous cue to provoke inflammation and promotes ESCC tumorigenesis. To the best of our knowledge, such a role for a ncRNA is unprecedented. All of our data here are new findings and thus leave many outstanding questions about nc886. The study of nc886 is just at the beginning stage and much more effort is needed for elucidation of its role and regulation in cancer, which should precede its clinical use.

## MATERIALS AND METHODS

### Cell lines and tissue samples

Cell lines in this study were obtained from Drs. Xiaochun Xu and Julie J. Izzo at the University of Texas MD Anderson Cancer Center, Houston, TX and cultured as described in Supplemental Information. Cell lines were validated by STR DNA fingerprinting using the AmpF_STR Identifiler kit (Applied Biosystems, Grand Island, NY) according to manufacturer's instructions. The STR profiles were compared to known ATCC fingerprints (http://www.atcc.org/) and to the Cell Line Integrated Molecular Authentication database (CLIMA, version 0.1.200808, http://bioinformatics.istge.it/clima/) [24]. The STR profiles matched known DNA fingerprints or were unique.

ESCC patients included in this study were those who had thoracic EC and underwent complete esophageal resection with adequate lymph node dissection, but without any perioperative treatment such as chemotherapy or radiotherapy. Tissues were collected freshly within 30 minutes after surgical removal and were stored at −196°C in the tumor bank at the National Cancer Center in Korea (NCC), after pathologist's review and macro-dissection. We chose 84 ESCC samples for which a tumor and its adjacent normal tissue were both present. Collection of human samples and protocols for investigation were approved by the Institutional Ethics Committee (No. NCCNCS-11-435) at NCC. Also, we obtained informed consent and agreement from patients. Epidemiological data were collected based on in-patient medical records in NCC.

### RNA isolation and measurement

Total RNA from patient tissue samples and cell lines was isolated by Trizol reagent (Invitrogen, Carlsbad, CA). Northern hybridization and qRT-PCR measurement of nc886 and other genes were performed as previously described [2]. Sequence information on qRT-PCR primers is available upon request.

### Pyrosequencing to measure CpG DNA methylation at the nc886 promoter region

Genomic DNA isolation and bisulfite-conversion were performed with a PureLink™ Genomic DNA kit (Invitrogen) and an EZ DNA methylation kit (Zymo Research, Orange, CA) respectively. Primers for pyrosequencing were as previously described [4].

### Statistical analysis of patient data

Kaplan-Meier plots and the log-rank test were used to estimate difference in patient's prognosis between two groups. When comparing two values, we used Student's t-test for continuous values and Chi-square or Fisher's exact test for discrete values. In analyzing nc886 expression and methylation data, the paired t-test was applied to evaluate the significance in difference between tumors and adjacent normal tissues. Correlation between nc886 expression and methylation was calculated by Spearman correlation analysis. All statistical tests were two-tailed, and a P-value less than 0.05 was considered to be significant.

### Reagents and antibodies

AzadC was purchased from Sigma-Aldrich (St. Louis, MO); CpG methyltransferase (M.Sssl) was from New England Biolabs (Ipswich, MA); Decade markers (= 10-nt ladder) for small RNA Northern and yeast tRNA were from Ambion (Carlsbad, CA); and protein and DNA size markers were from GenDepot (Barker, TX). The source of antibodies was described in [1, 2].

### *In vitro* methylated DNAs, and RNAs, transfection, and assays

PCR amplification of “649-mer DNA” (illustrated in Fig 2A and used in Fig 2F) and its *in vitro* methylation and transfection was elaborated in Supplemental Information. Anti-oligos (“anti886 75-56” and “anti-vt 21-2”), siRNA against PKR, and *in vitro* transcribed RNA (nc886 and vtRNA1-1) were prepared as previously described [1, 2, 8]. Lipofectamine™ RNAiMAX reagent (Invitrogen) was used for transfection of anti-oligos; Lipofectamine™ 2000 reagent (Invitrogen) was used for 649-mer DNA and *in vitro* transcribed RNA. Detailed transfection protocols are described in Supplemental Information. Luciferase reporter assays and cell proliferation assays were performed as described previously [2]. The AP-1 reporter plasmid (a kind gift from Dr. Xiaoyong Bao at the University of Texas Medical Branch, Galveston, TX) encodes a firefly luciferase gene whose expression is driven by three copies of AP-1 recognition elements originally isolated from the IL-8 promoter.

### mRNA microarray and pathway analysis

Transfection of “anti886 75-56” and “anti-vt 21-2” (for nc886 knockdown and control respectively) was performed in triplicate. Briefly, probe preparation and array run were done by using a TotalPrep™ RNA amplification kit and a HumanHT-12 v4.0 Expression BeadChip kit (Illumina, San Diego, CA) per the manufacturer's instructions. A more detailed protocol is described in Supplemental Information. The array data were deposited in the Gene Expression Omnibus (accession number GSE51732; Reviewer's link, “http://www.ncbi.nlm.nih.gov/geo/query/acc.cgi?token=ulergucevhsxbqp&acc=GSE51732”). For gene set and pathway analysis from the gene expression data, we used PAGE (Parametric Analysis of Gene set Enrichment) method with MSigDB (ver 3.0) gene sets [25, 26].

## SUPPLEMENTARY INFORMATION


