# Supplementary material for: Epigenetic silencing of the non-coding RNA nc886 provokes oncogenes during human esophageal tumorigenesis
Source: Oncotarget. 2014 Apr 27;5(11):3472–81. doi: 10.18632/oncotarget.1927 (PMC4116496; doi:10.18632/oncotarget.1927)

SUPPLEMENTAL INFORMATION

(for Lee H-S and Lee K, *et al*. "**Epigenetic silencing of the non-coding RNA nc886 provokes oncogenes during human esophageal tumorigenesis**")

SUPPLEMENTAL MATERIALS AND METHODS

Cell culture

A non-malignant esophageal cell line Het-1A was maintained in Keratinocyte-SFM (serum-free medium) containing 5 ng/ml human recombinant epidermal growth factor (Sigma-Aldrich), 0.05 mg/ml bovine pituitary extract (Invitrogen), 0.005 mg/ml human recombinant insulin (Invitrogen), and 500 ng/ml hydrocortisone (Sigma-Aldrich). BE-3 and TT cell lines were maintained in DMEM medium; TE-1, TE-8, TE-12, SK-4, OE-33 cell lines were in RPMI-1640. DMEM and RPMI-1640 medium were supplemented with 10% fetal bovine serum and 1% penicillin/streptomycin. Cells were cultured in a standard CO_2 incubator at 37°C.

Preparation and transfection of *in vitro* methylated DNA

A genomic DNA fragment harboring nc886 and its flanking sequences ("649-mer DNA" illustrated in Fig 2A) was PCR-amplified on human genomic DNA isolated from a cholangiocarcinoma cell line M139. The forward and reverse primers are respectively 5'-CTGCTGGACCTAGGTAGACG-3' and 5'-AATCCATAACGCACTCCGCG-3'. The amplified DNA was confirmed by sequencing and *in vitro* methylated by M.Sssl per manufacturer's instruction. After M.Sssl (or no enzyme control) treatment, proper methylation was assured by digesting methylated 649-mer DNA (or unmethylated control) with methylation-sensitive restriction enzymes *Hha*I/*Hpa*II (data not shown). These DNAs were transfected to TT cells and cells were harvested at 24 hrs for Northern hybridization of nc886 and 5S rRNA in Fig 2F. In addition, we recovered soluble DNA from the aqueous phase during Trizol RNA preparation and measured nc886 DNA fragments by PCR using two primers (5'-GTTTCAGTCGCACACTCCTACC-3' and 5'-AAGGGTCAGTAAGCACCCGCG-3'). In Fig 2F, the amplified bands were of a nearly identical intensity between lanes 2-3, indicating similar transfection efficiencies between methylated and unmethylated DNA. By titrating input DNA amounts in PCR reactions, we ascertained that the similarly intense bands were not due to saturation of PCR amplification. The PCR product was from the transfected DNA but not from contaminating genomic DNA or RNA, as indicated by no PCR product in the untransfected sample (lane 1 in Fig 2F) and disappearance of the PCR product by DNase treatment prior to PCR.

Transfection of anti-oligos for nc886 knockdown

During nc886 knockdown, "anti-vt 21-2" targeting vault RNAs was used as a negative control, because vault RNAs are neither suppressed in cancer nor affect cell proliferation upon knockdown although vtRNAs are of similar abundance and length to nc886 [1, 2]. We

used Lipofectamine™ RNAiMAX reagent (Invitrogen) for transfection of these anti-oligos. A forward transfection protocol (a transfection mixture added to pre-plated cells) was used for TE-1 and TE-8 cells; a reverse transfection protocol (cells plated after mixing with a transfection mixture) was for Het-1A cells. Cells were harvest at 24 hrs post-transfection.

mRNA array

Prior to array run, isolated total RNA (by Trizol reagent) was subjected to integrity and quality check by using the Bioanalyzer with Experion RNA Stdsens analysis kit and the software (Bio-Rad, Montreal, Quebec. Canada). 750 ng of total RNA was used for labeling and hybridization according to the manufacturer's protocols (Illumina®). After bead chips were scanned with a BeadArray Reader (Illumina®), microarray data were normalized using the quantile normalization method in the Linear Models for Microarray Data package in the R language environment [3]. The expression level of each gene was log_2-transformed before further analysis. The random-variance *t* test was used to identify genes differentially expressed between the two classes, which were compared using BRB ArrayTools [4]. The random-variance *t* test is an improvement over the standard separate *t* test as it allows information to be shared among genes about within-class variation without assuming that all genes have the same variance. Gene expression differences were considered significant if $P < 0.001$.

Transfection of *in vitro* transcribed RNA

Cells were mixed with each *in vitro* transcribed RNA combined with Lipofectamine™ 2000 reagent and then plated to at 35-50% confluency. The amount of total RNA in a transfection mixture was adjusted to 120 ng (per 96-well) by adding yeast tRNA and kept constant in all the experiments.

SUPPLEMENTAL REFERENCES

1. Lee K, Kunkeaw N, Jeon SH, Lee I, Johnson BH, Kang GY, Bang JY, Park HS, Leelayuwat C and Lee YS. Precursor miR-886, a novel noncoding RNA repressed in cancer, associates with PKR and modulates its activity. RNA. 2011; 17(6):1076-1089.

2. Kunkeaw N, Jeon SH, Lee K, Johnson BH, Tanasanvimon S, Javle M, Pairojkul C, Chamgramol Y, Wongfieng W, Gong B, Leelayuwat C and Lee YS. Cell death/proliferation roles for nc886, a non-coding RNA, in the protein kinase R pathway in cholangiocarcinoma. Oncogene. 2012.

3. Bolstad BM, Irizarry RA, Astrand M and Speed TP. A comparison of normalization methods for high density oligonucleotide array data based on variance and bias. Bioinformatics. 2003; 19(2):185-193.

4. Qi Q, Zhao Y, Li M and Simon R. Non-negative matrix factorization of gene expression profiles: a plug-in for BRB-ArrayTools. Bioinformatics. 2009; 25(4):545-547.

SUPPLEMENTAL FIGURE AND TABLE LEGENDS

Figure S1. nc886 is a 101 nts ncRNA, but not a miRNA

The whole Northern blots for nc886 (Fig 1A) and ethidium bromide staining of the gels. All descriptions are the same as Fig 1A and D.

Figure S2. Poorer survival of ESCC patients with lower nc886 expression

A-B. Kaplan-Meier curves for recurrence-free survival (RFS in panel A) and overall survival (OS in panel B) according to nc886 expression and pathological staging. The + symbols in the panel indicate censored data.

Table S1. Status of nc886 expression level in 84 ESCC tumors with respect to clinico-pathological characteristics

All tumors were classified into two sub-groups according to nc886 RNA expression levels (which are “Tumor/Normal” values calculated as described in Fig 1A). The median value was used to discriminate the two groups. *A P-value less than 0.05 was considered significant. Between nc886 low- and high- groups, a significant difference was seen in pathologic TNM stage (according to AJCC/UICC 7th edition). Low nc886 expression was associated with more advanced stage III. No significant difference was found in the other variables.

Figure S3. Heapmap of the 33 commonly induced genes and nc886 in mRNA array data

Heatmap showing the 33 genes (top 33 rows) and nc886 (the bottom row). nc886 was the most reduced gene in all the three cell lines. Clustering of triplicate samples from each of the three cell lines (transfected with “anti886 75-56” or “anti-vt 21-2”) is also shown.

Figure S4. Intersection of induced and reduced genes upon nc886 knockdown among the three cell lines

Venn diagram of induced (> 0.5 fold) and reduced (< -0.5 fold) genes in panel A and B respectively.

Table S2. List of 33 induced genes upon nc886 knockdown

As shown in Fig 3B (also in Fig S3 and S4A), 33 genes were induced (> 0.5 fold) commonly in HET-1A, TE-1, and TE-8. These genes are tabulated in alphabetical order. Expression values are an average of triplicates.

Figure S5. Intersection of induced and reduced pathways and transcription factor targets upon nc886 knockdown among the three cell lines

Venn diagram of BIOCARTA pathways (panel A) and transcription factor targets (panel B).

Fig S1

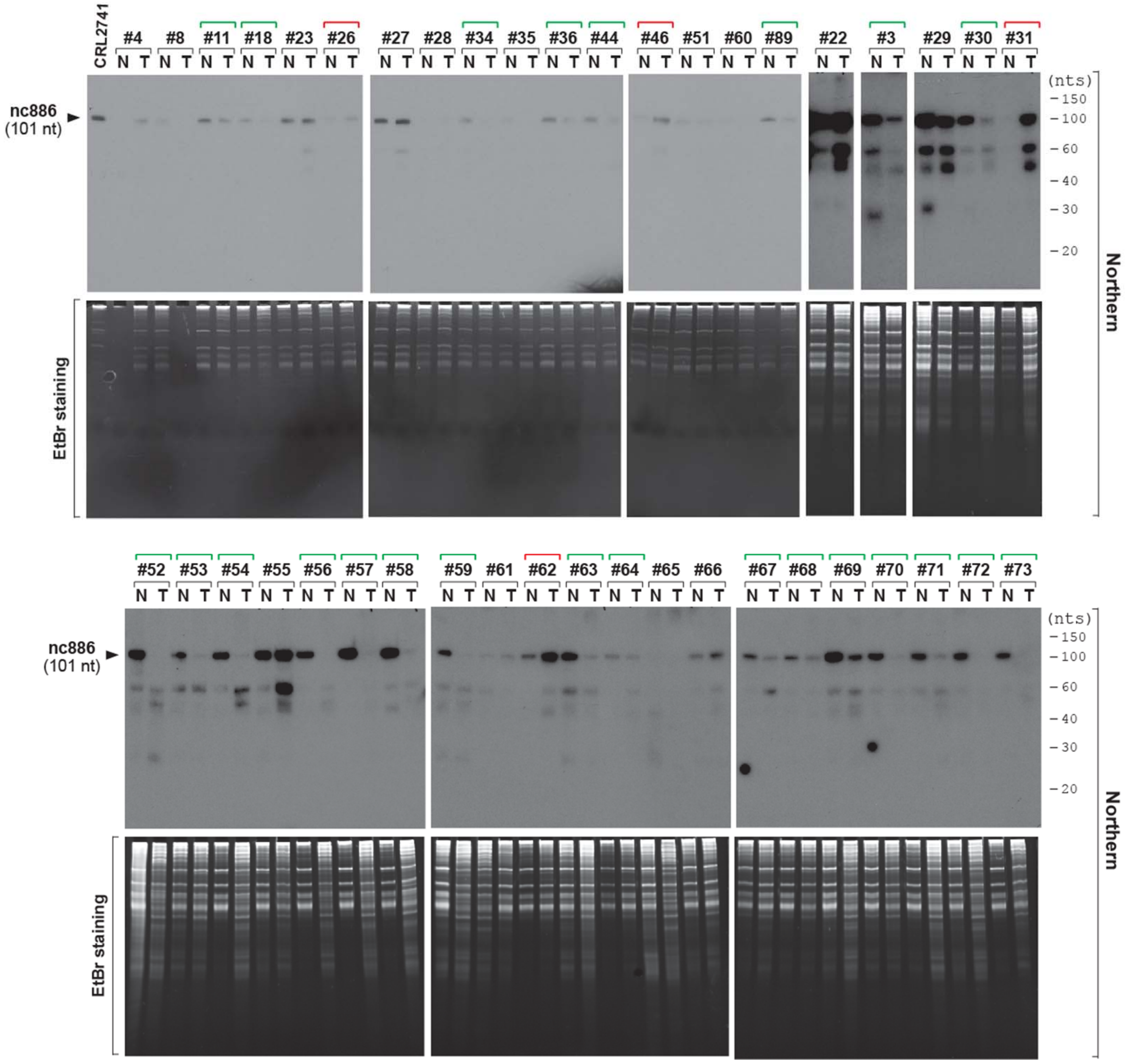

CRL2741
#4
#8
#11
#18
#23
#26
#27
#28
#34
#35
#36
#44
#46
#51
#60
#89
#22
#3
#29
#30
#31
N T
nc886
(101 nt)
(nts)
150
100
60
40
30
20
Northern
EtBr staining
#52
#53
#54
#55
#56
#57
#58
#59
#61
#62
#63
#64
#65
#66
#67
#68
#69
#70
#71
#72
#73

Fig S1 (continued)

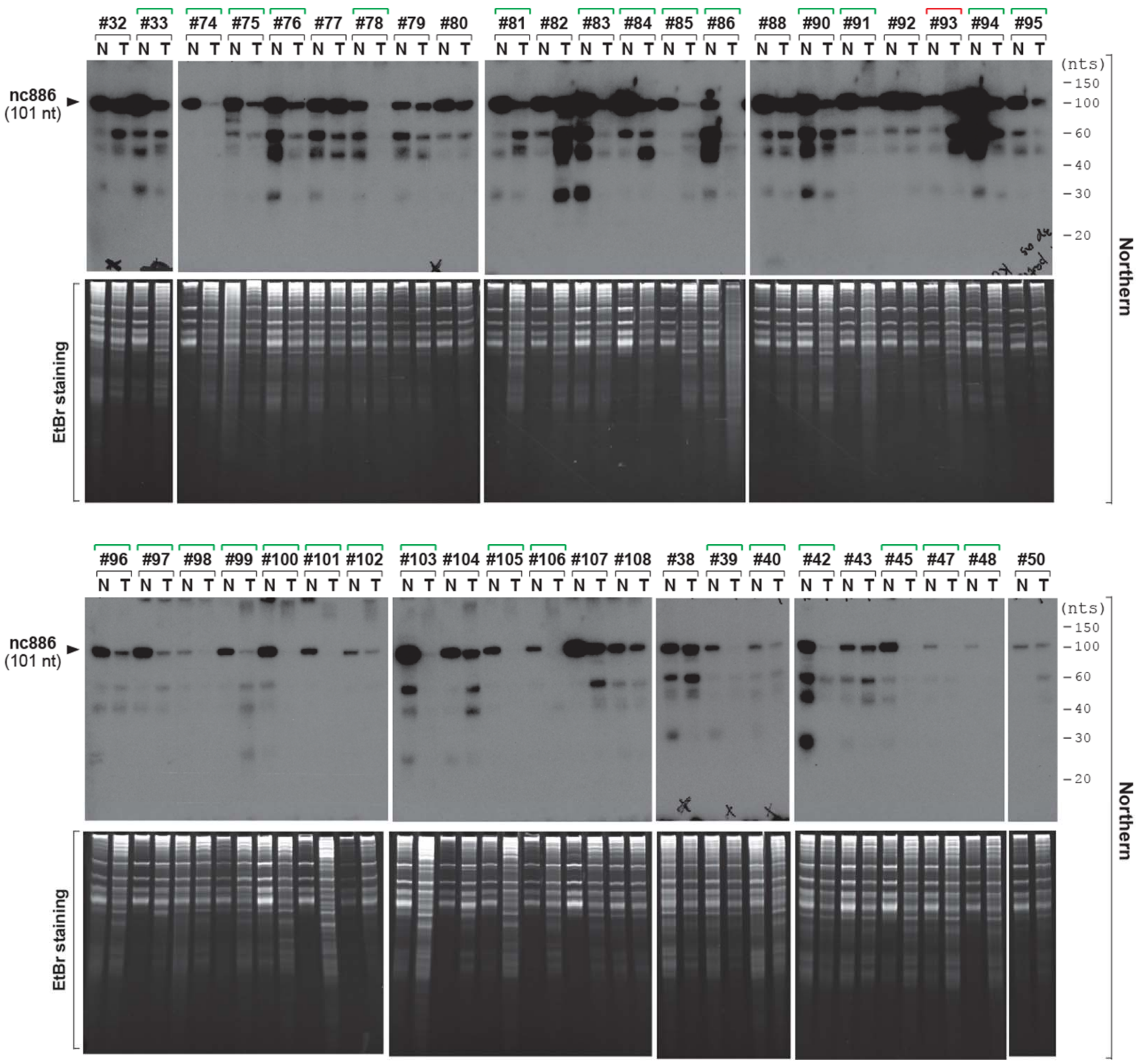

Fig S2

A

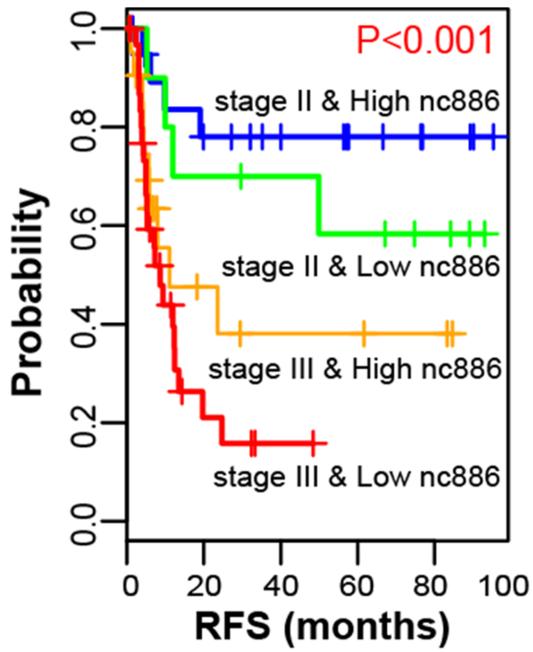
P<0.001
stage II & High nc886
stage II & Low nc886
stage III & High nc886
stage III & Low nc886
Probability
RFS (months)

B

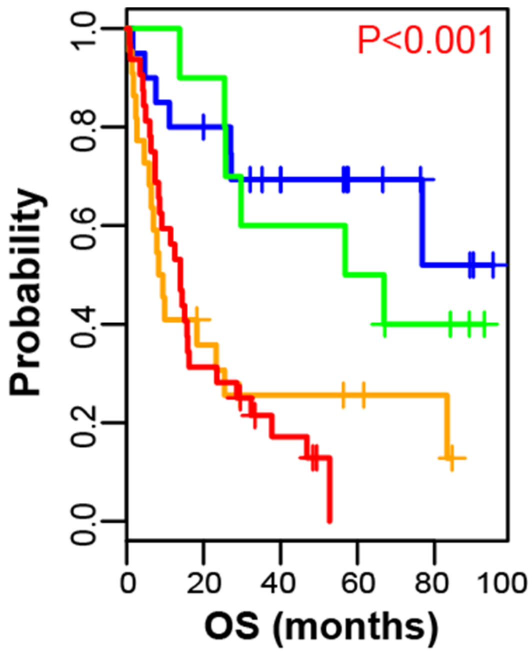
P<0.001
Probability
OS (months)

Table S1

Variable		High nc886	Low nc886	*p*-value[*]
Number of patients		42	42	
Sex	Men	39 (92.9%)	40 (95.2%)	1.0
	Women	3 (7.1%)	2 (4.8%)	
Age (years)		66.9 ± 6.9	65.2 ± 5.6	0.19
Smoking		22 (52.4%)	31 (73.8%)	0.07
Pathologic T status	1	5 (11.9%)	0 (0%)	0.09
	2	3 (7.1%)	6 (14.3%)	
	3	32 (76.2%)	32 (76.2%)	
	4	2 (4.8%)	4 (9.5%)	
Pathologic N status	0	19 (45.2%)	11 (26.2%)	0.26
	1	13 (31.0%)	18 (42.9%)	
	2	6 (14.3%)	10 (23.8%)	
	3	4 (9.5%)	3 (7.1%)	
Pathologic TNM stage	II	20 (47.6%)	10 (23.8%)	0.04
	III	22 (52.4%)	32 (76.2%)	
Postoperative Complications		24 (57.1%)	20 (47.6%)	0.51
Median follow-up (month)		32.8	27.0	0.34

Fig S3

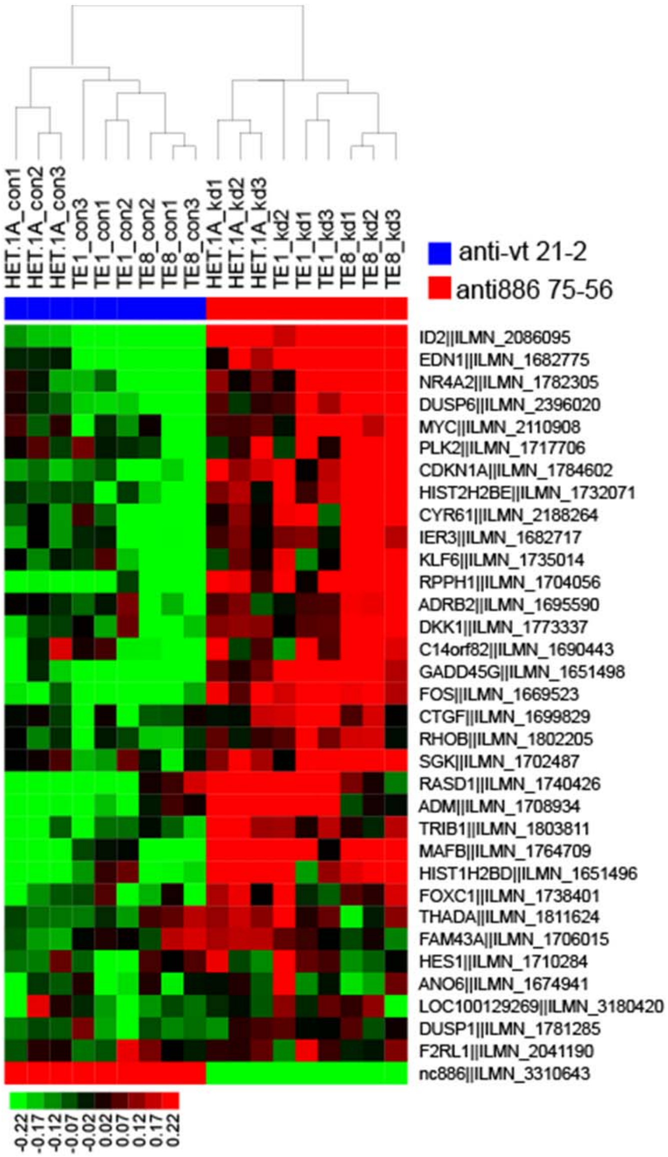

HET.1A_con1
HET.1A_con2
HET.1A_con3
TE1_con3
TE1_con1
TE1_con2
TE8_con2
TE8_con1
TE8_con3
HET.1A_kd1
HET.1A_kd2
HET.1A_kd3
TE1_kd2
TE1_kd1
TE1_kd3
TE8_kd1
TE8_kd2
TE8_kd3
anti-vt 21-2
anti886 75-56
ID2||ILMN_2086095
EDN1||ILMN_1682775
NR4A2||ILMN_1782305
DUSP6||ILMN_2396020
MYC||ILMN_2110908
PLK2||ILMN_1717706
CDKN1A||ILMN_1784602
HIST2H2BE||ILMN_1732071
CYR61||ILMN_2188264
IER3||ILMN_1682717
KLF6||ILMN_1735014
RPPH1||ILMN_1704056
ADRB2||ILMN_1695590
DKK1||ILMN_1773337
C14orf82||ILMN_1690443
GADD45G||ILMN_1651498
FOS||ILMN_1669523
CTGF||ILMN_1699829
RHOB||ILMN_1802205
SGK||ILMN_1702487
RASD1||ILMN_1740426
ADM||ILMN_1708934
TRIB1||ILMN_1803811
MAFB||ILMN_1764709
HIST1H2BD||ILMN_1651496
FOXC1||ILMN_1738401
THADA||ILMN_1811624
FAM43A||ILMN_1706015
HES1||ILMN_1710284
ANO6||ILMN_1674941
LOC100129269||ILMN_3180420
DUSP1||ILMN_1781285
F2RL1||ILMN_2041190
nc886||ILMN_3310643
-0.22
-0.17
-0.12
-0.07
-0.02
0.02
0.07
0.12
0.17
0.22

Fig S4

A

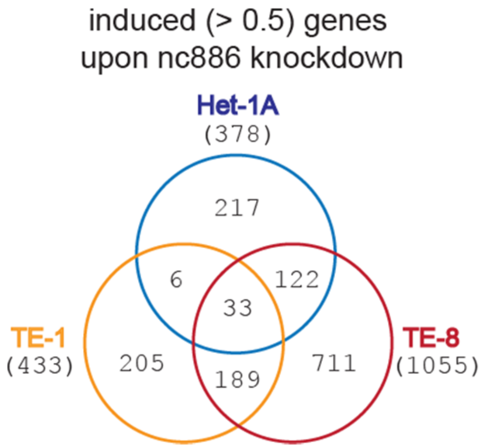
induced (> 0.5) genes
upon nc886 knockdown
Het-1A
(378)
TE-1
(433)
TE-8
(1055)
217
6
122
33
205
189
711

B

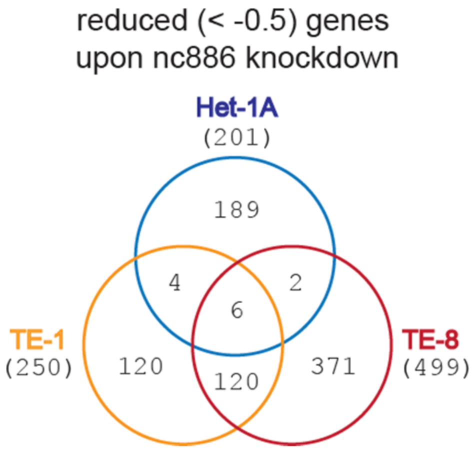
reduced (< -0.5) genes
upon nc886 knockdown
Het-1A
(201)
TE-1
(250)
TE-8
(499)
189
4
2
6
120
120
371

Table S2

			Het-1A			TE-1			TE-8		
GeneID	SYMBOL	SEARCH_KEY	ctrl	kd	**kd / ctrl**	ctrl	kd	**kd / ctrl**	ctrl	kd	**kd / ctrl**
ILMN_1708934	**ADM**	NM_001124.1	10.421	11.497	**1.076**	11.025	12.046	**1.021**	12.484	13.199	**0.716**
ILMN_1695590	**ADRB2**	NM_000024.3	8.287	8.791	**0.505**	7.934	8.441	**0.507**	7.779	8.978	**1.199**
ILMN_1674941	**ANO6**	NM_001025356.1	8.159	8.732	**0.573**	9.030	9.744	**0.714**	8.592	9.327	**0.734**
ILMN_1690443	**C14orf82**	XM_944991.1	10.472	11.157	**0.684**	9.379	9.963	**0.584**	9.693	11.004	**1.310**
ILMN_1784602	**CDKN1A**	NM_078467.1	10.103	10.888	**0.785**	10.780	11.652	**0.872**	8.917	10.342	**1.425**
ILMN_1699829	**CTGF**	NM_001901.1	12.517	13.045	**0.528**	9.911	10.894	**0.983**	7.414	8.295	**0.881**
ILMN_2188264	**CYR61**	NM_001554.3	10.969	11.554	**0.585**	10.498	11.251	**0.752**	8.605	10.164	**1.559**
ILMN_1773337	**DKK1**	NM_012242.2	11.189	11.868	**0.679**	12.063	12.599	**0.536**	11.198	12.454	**1.256**
ILMN_1781285	**DUSP1**	NM_004417.2	10.947	11.479	**0.532**	11.333	11.922	**0.588**	8.756	9.605	**0.849**
ILMN_2396020	**DUSP6**	NM_022652.2	10.736	11.252	**0.516**	9.254	10.088	**0.834**	10.426	11.943	**1.517**
ILMN_1682775	**EDN1**	NM_001955.2	8.311	8.939	**0.628**	9.537	10.769	**1.232**	8.224	10.155	**1.930**
ILMN_2041190	**F2RL1**	NM_005242.3	9.620	10.139	**0.519**	9.800	10.306	**0.506**	10.350	11.097	**0.747**
ILMN_1706015	**FAM43A**	NM_153690.4	8.805	9.542	**0.738**	7.606	8.109	**0.503**	8.684	9.287	**0.603**
ILMN_1669523	**FOS**	NM_005252.2	8.041	8.871	**0.830**	7.657	8.756	**1.099**	7.565	8.771	**1.206**
ILMN_1738401	**FOXC1**	NM_001453.1	9.812	10.541	**0.728**	12.164	12.780	**0.616**	11.004	11.978	**0.974**
ILMN_1651498	**GADD45G**	NM_006705.2	9.526	10.220	**0.695**	8.401	10.345	**1.944**	7.130	8.645	**1.515**
ILMN_1710284	**HES1**	NM_005524.2	7.766	8.276	**0.510**	8.531	9.456	**0.925**	9.197	9.835	**0.638**
ILMN_1651496	**HIST1H2BD**	NM_138720.1	9.499	10.565	**1.066**	10.205	10.762	**0.557**	7.858	9.117	**1.259**
ILMN_1732071	**HIST2H2BE**	NM_003528.2	7.764	8.367	**0.603**	8.652	9.443	**0.791**	7.557	8.785	**1.227**
ILMN_2086095	**ID2**	NM_002166.4	7.787	8.645	**0.858**	6.851	8.276	**1.425**	9.135	11.076	**1.941**
ILMN_1682717	**IER3**	NM_052815.1	10.296	10.903	**0.606**	12.083	12.714	**0.631**	10.718	12.025	**1.307**
ILMN_1735014	**KLF6**	NM_001300.4	10.723	11.244	**0.520**	11.710	12.260	**0.550**	10.652	12.164	**1.512**
ILMN_3180420	**LOC100129269**	XM_001719843.1	12.202	12.724	**0.523**	8.886	9.596	**0.710**	10.427	11.235	**0.808**
ILMN_1764709	**MAFB**	NM_005461.3	8.296	10.036	**1.740**	7.150	7.863	**0.713**	7.519	9.174	**1.655**
ILMN_2110908	**MYC**	NM_002467.3	9.687	10.208	**0.521**	10.456	11.338	**0.883**	11.204	12.395	**1.191**
ILMN_1782305	**NR4A2**	NM_006186.2	8.024	8.603	**0.579**	7.263	8.090	**0.826**	8.100	9.809	**1.709**
ILMN_1717706	**PLK2**	NM_006622.1	8.676	9.203	**0.527**	8.961	9.500	**0.539**	8.559	9.917	**1.358**
ILMN_1740426	**RASD1**	NM_016084.3	8.062	9.677	**1.615**	7.065	8.391	**1.326**	7.033	7.706	**0.673**
ILMN_1802205	**RHOB**	NM_004040.2	9.465	10.011	**0.546**	10.071	10.828	**0.758**	9.652	10.653	**1.001**
ILMN_1704056	**RPPH1**	NR_002312.1	11.179	12.047	**0.869**	11.089	12.045	**0.955**	9.681	12.824	**3.143**
ILMN_3229324	**SGK1**	NM_005627.3	8.772	9.363	**0.591**	7.917	9.059	**1.142**	8.496	9.384	**0.888**
ILMN_1811624	**THADA**	NM_198554.1	9.025	9.712	**0.687**	7.490	8.213	**0.723**	7.564	8.176	**0.612**
ILMN_1803811	**TRIB1**	NM_025195.2	8.742	9.689	**0.947**	9.855	10.571	**0.716**	9.763	10.659	**0.896**

Fig S5

A

induced BIOCARTA pathways upon nc886 knockdown (cutoff Z-score = 3.0)

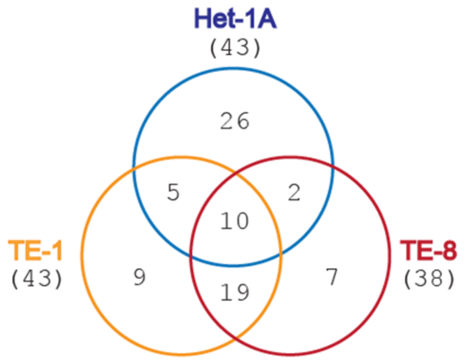

reduced BIOCARTA pathways upon nc886 knockdown (cutoff Z-score = -3.0)

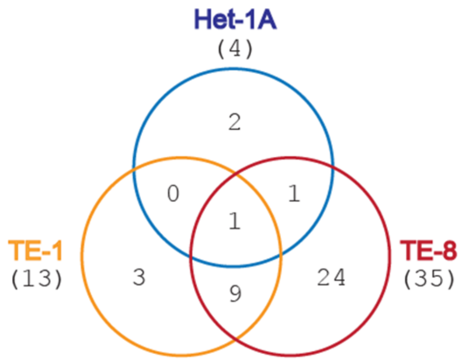

B

induced TFT (Transcription Factor Targets) upon nc886 knockdown (cutoff Z-score = 3.0)

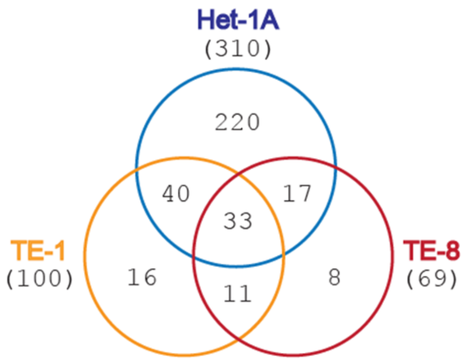

reduced TFT (Transcription Factor Targets) upon nc886 knockdown (cutoff Z-score = -3.0)

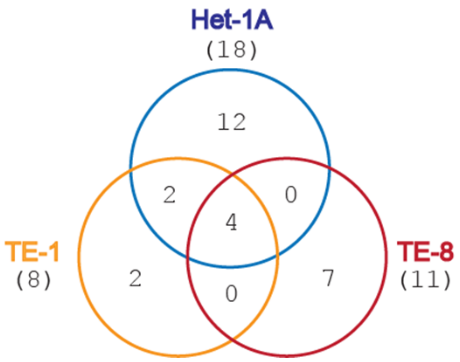

Supplement: Supplementary file 1 [file oncotarget-05-3472-s001.pdf]
